# Contribution of classification based on ferroptosis-related genes to the heterogeneity of MAFLD

**DOI:** 10.1186/s12876-022-02137-9

**Published:** 2022-02-10

**Authors:** Xin Dai, Rui Zhang, Bangmao Wang

**Affiliations:** 1 Department of Gastroenterology and Hepatology, General Hospital, Tianjin Medical University, Tianjin, 300052 China; 2 Department of Nosocomial Infection, The Forth Central Hospital of Tianjin, Tianjin, 300140 China

**Keywords:** Metabolic dysfunction-associated fatty liver disease, Ferroptosis, Heterogeneity, Immune status, Subtypes

## Abstract

**Background:**

Metabolic dysfunction-associated fatty liver disease (MAFLD) is a highly heterogeneous disease and its heterogeneity might be associated with ferroptosis because ferroptosis plays an important role in the development of MAFLD. We aimed to perform integrative analysis of ferroptosis related genes and MAFLD subtypes using bioinformatics.

**Methods:**

A differential expression analysis was performed to identify key ferroptosis-related genes associated with the clinical characteristics of MAFLD. Furthermore, consensus k clustering was utilized to distinguish ferroptosis-related clinical subtypes of MAFLD and assess the association of ferroptosis-related gene expression and clinical features between patients with different subtypes of MAFLD. Moreover, the variation in the immune status and regulatory relationship of ferroptosis-related genes in individuals with MAFLD was also explored using single sample gene set enrichment analysis, weighted gene coexpression network analysis and enrichment analyses.

**Results:**

Eight ferroptosis-related genes were identified as closely associated with both the hepatic steatosis grade and non-alcoholic fatty liver disease activity score. Two subtypes of MAFLD based on ferroptosis-related genes were identified by consensus clustering. They exhibited significantly different clinical features, immune statuses, biological processes and outcomes. The progression of the two subtypes was associated with immunity.

**Conclusions:**

Two highly heterogeneous subtypes of MAFLD with significantly distinct clinical features, biological processes and immune statuses were identified based on ferroptosis-associated genes, which strongly supports the hypothesis that ferroptosis plays an important role in the development of MAFLD.

**Supplementary Information:**

The online version contains supplementary material available at 10.1186/s12876-022-02137-9.

## Introduction

Recently, a growing consensus has been established among experts that metabolic dysfunction-associated fatty liver disease (MAFLD) is suggested a more proper umbrella term to replace the original term nonalcoholic fatty liver disease (NAFLD). One of the most important reasons for this change in nomenclature is that the outdated NAFLD acronym lacks an adequate consideration of heterogeneity, which represents a major obstacle to the discovery of highly effective drug therapy [1]. MAFLD is a highly heterogeneous disease with a natural history and a broad spectrum of disease severity, along with considerable interpatient variability across the spectrum. For instance, hepatic steatosis is extremely widespread, but only a small number of patients exhibit inflammation [2]. Even in patients with steatohepatitis, the progression of liver fibrosis is significantly accelerated in some patients, but is slow in other patients [3].

Previously, patients with MAFLD were divided into two subtypes in clinical practice, including those with steatohepatitis and others without. However, its reasonableness is a matter of debate. Experts have questioned whether this classification fully recapitulates the pathological development of MAFLD. In addition, it does not reveal the mechanism of different subtypes [1]. Therefore, a new classification for MAFLD to facilitate the elucidation of its pathogenesis is needed.

The mechanisms driving MAFLD progression include abnormal accumulation of lipid droplets in hepatocytes, oxidative stress, inflammatory cell infiltration, increased hepatocyte cell death and subsequent hepatic fibrosis. Different types of cell death, such as necroptosis, pyroptosis and ferroptosis, coexist as metabolic liver disease progresses [4]. Ferroptosis is an iron-dependent form of regulated cell death, which is characterized by the excess production of reactive oxygen species and overwhelming lipid peroxidation [5]. In recent studies, ferroptosis has been recognized to play an important role in the development of MAFLD [6]. Treatment with RSL-3, a ferroptosis inducer, aggravates hepatic steatosis, inflammatory and serum biochemical levels in methionine/choline-deficient diet-fed mice. Administration of deferoxamine mesylate salt significantly reduces the MAFLD severity and eliminates the harmful effects of RSL-3 on methionine/choline-deficient diet-fed mice [7]. In addition, previous studies have revealed that ferroptosis is closely related to tumor heterogeneity [8]. Nevertheless, the mechanism by which ferroptosis mediates the heterogeneity of MAFLD remains largely unknown. Fortunately, a large amount of MAFLD-related transcriptome data are available in public databases, while bioinformatics analysis is becoming increasingly powerful and more widely used, helping us to perform integrative analyses of ferroptosis- related genes and MAFLD molecular subtypes.

In this study, we systematically analyzed the expression of widely reported ferroptosis- associated genes in individuals with MAFLD using the public Gene Expression Omnibus (GEO) database. First, we performed a differential expression analysis to identify the critical ferroptosis-related genes associated with the clinical characteristics of MAFLD. Furthermore, we explored the subtypes of MAFLD based on ferroptosis-related genes using unsupervised clustering. In addition, we attempted to explore the explanations for distinct clinical features in ferroptosis-related gene-associated subtypes by performing an enrichment analysis.

## Methods

### Study selection and data sources

The publicly available GEO database (https://www.ncbi.nlm.nih.gov/geo/) was searched for all expression microarrays that matched the term metabolic dysfunction-associated fatty liver disease or MAFLD. The inclusion criteria included i) patients aged 18 years or older, ii) diagnosed with MAFLD based on liver biopsy, and iii) preservation of clinical indices such as NAFLD activity score (NAS) and the grade of liver steatosis.

Dataset GSE130970 was downloaded for further analysis. In addition, Dataset GSE89632 was selected for verification. Ferroptosis-related genes were prescreened according to the published literature in the PubMed database (https://pubmed.ncbi.nlm.nih.gov/). The differential expression analysis was performed in the GSE130970 dataset, and genes associated with the clinical characteristics of MAFLD in this dataset were obtained. The Corrplot package was utilized to validate the interrelationships among the 8 genes with Pearson’s correlation analysis.

### Exploration of the subtypes of MAFLD based on ferroptosis-related genes

Consensus k clustering was utilized to perform consistent clustering of the ferroptosis-related gene expression profiles of the 78 patients with MAFLD in the GSE130970 dataset. Clustering was performed using 50 repetitions, with each repetition involving 80% of the samples. The optimal cluster number distinguished the subtypes of MAFLD was determined based on cumulative distribution function curves of the consensus score and clear separation of the consensus matrix heatmaps. Principal component analysis (PCA), which can replace the original mass of variables with fewer comprehensive variables to plot the similarities and differences between samples, was performed. The mRNA expression of the 8 ferroptosis related gene above was utilized to calculate first principal component PC1 and second principal component PC2, verifying differences in distribution between subtypes.

### Assessment of the correlations of ferroptosis-related gene expression with clinical features between patients with different subtypes of MAFLD

The pheatmap package was utilized to generate the ferroptosis-related gene expression profiles and clinical features of the two subtypes of MAFLD. The tableone package was applied for the statistical analysis of the clinical features. A two-sided test was used, and a p value < 0.05 was considered significant.

### Assessment of immune heterogeneity between subtypes

We further evaluated the variation in immune status between patients with different subtypes of MAFLD. Single sample gene set enrichment analysis (ssGSEA) and cell-type identification by estimating relative subsets of RNA transcript (CIBERSORT) were used to evaluate the infiltration of immune cells in each sample in the GSE130970 dataset. Briefly, we used GSVA and the GSEABase R package to calculate separate enrichment scores for each pair of a sample and gene set [9]. Each GSEA enrichment score represented the degree to which the genes in a particular gene set were coordinately up- or downregulated within a sample. CIBERSORT, a computational method, is a deconvolution algorithm that calculates the fractions of immune cells according to the RNA matrix (https://cibersort.stanford.edu/) [10, 11]. We used the R package “CIBERSORT” to estimate the fraction of immune cells in GSE130970 samples. We used Spearman’s correlation analysis to analyze the correlations between ferroptosis-related gene expression and the proportions of immune cells. Additionally, immune cell types were identified and proportions were calculated. Immune cells included immunosuppressive cells such as Treg cells and Th2 cells, as well as immune-enhancing cells such as activated CD4 T cells, Th1 cells, activated B cells and activated NK cells.

### Weighted gene coexpression network analysis (WGCNA)

We performed WGCNA to detect coexpressed gene modules and explore the associations between genes and phenotypes, as well as the hub genes in the gene network [12]. We constructed unsigned coexpression networks based on 10,000 genes with the greatest variations in expression using the WGCNA package in R. The soft threshold was 9. Afterward, the adjacency matrix was transformed into the topological overlap matrix. The purpose of this procedure is to introduce the interconnections between genes. We applied hierarchical clustering to cluster the gene trees. The module from the system clustering tree was identified using the dynamic cutting algorithm. Different colors represented different modules. According to the weighted correlation coefficient, genes were classified according to their expression patterns. Genes with similar patterns were grouped into a module.

### Statistical analysis

The experimental results are shown as the means ± SEM for three separate experiments. Student’s t test was performed and data were subsequently analyzed using R language (version 3.6). A two-tailed P value < 0.05 was considered significant.

## Results

### MAFLD datasets and ferroptosis-related genes

**Fig. 1:**
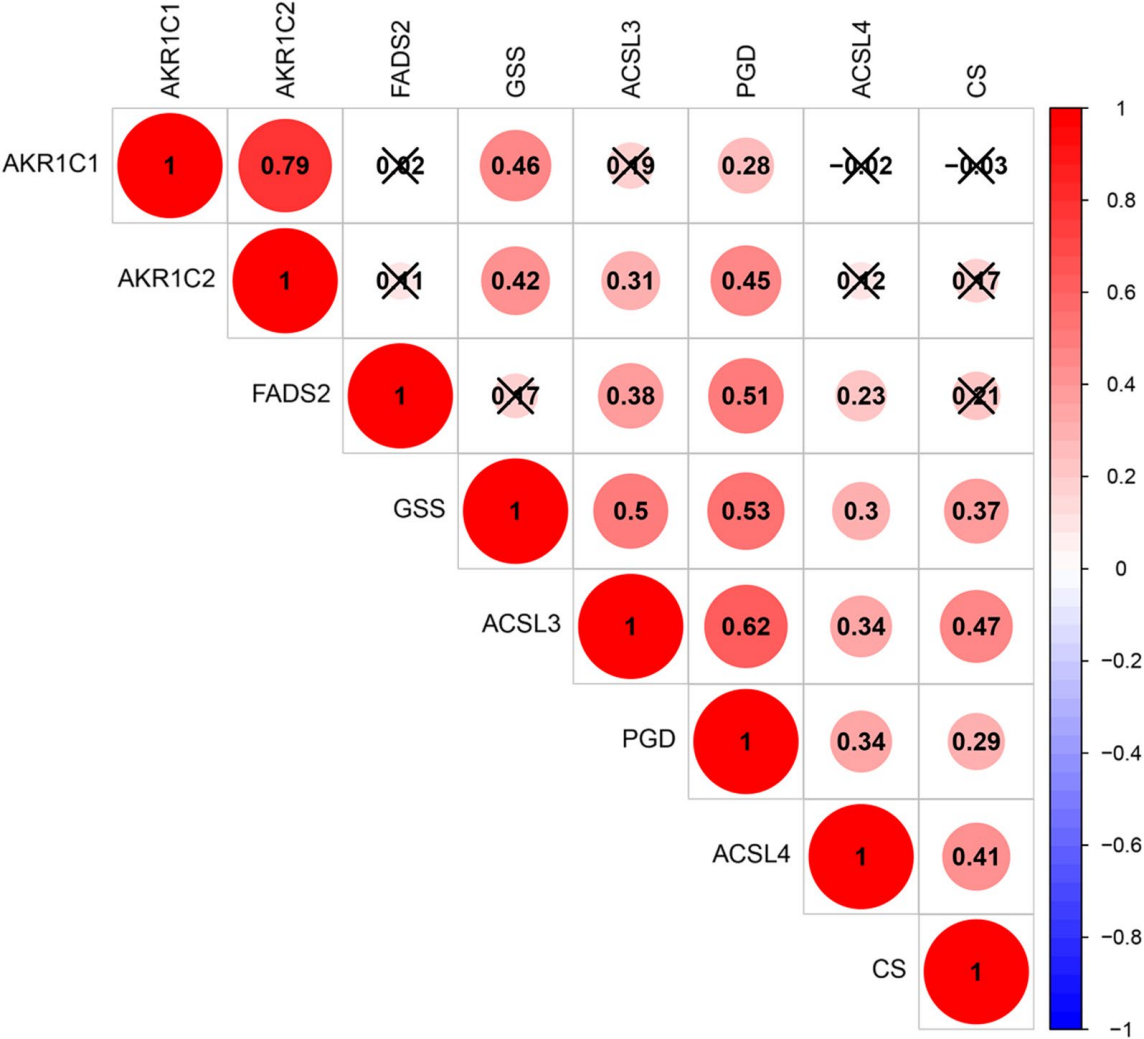
The correlations among 8 ferroptosis-related genes. The number in the plots represents the Pearson correlation coefficient. × represents P > 0.05

Based on the retrieval tactics and inclusion criteria, dataset GSE130970 was finally included in the current study. Seventy-eight patients with MAFLD in this dataset were all diagnosed through liver biopsy. Information on their transcriptomic data and clinical features, such as hepatic steatosis grade and NAS was completely preserved. We first collected 60 genes related to ferroptosis according to the published literature in the PubMed database and then investigated the differential expression of ferroptosis-related genes among patients with different clinical features. This analysis yielded 8 ferroptosis-related genes that were closely related to both the hepatic steatosis grade and NAS. These ferroptosis related genes included acyl-CoA synthetase long-chain family member 3(ACSL3), acyl-CoA synthetase long-chain family member 4(ACSL4), aldo–keto reductase family 1 member C1(AKR1C1), aldo–keto reductase family 1 member C2 (AKR1C2), citrate synthase (CS), fatty acid desaturase 2 (FADS2), glutathione synthetase (GSS) and phosphoglycerate dehydrogenase (PGD). Details are shown in Table 1. A triangle heatmap showed that these 8 genes were closely related to each other (Fig. 1). The classification of the subtypes of MAFLD according to the 8 ferroptosis-related genes was reliable.

**Table 1:** Eight ferroptosis-related genes analyzed in this study

	Steatosis grade	NAS
ACSL3	7.935 (0.047)	14.152 (0.028)
ACSL4	14.715 (0.002)	23.841 (5.585e−04)
AKR1C1	8.568 (0.036)	12.816 (0.046)
AKR1C2	14.943 (0.002)	15.474 (0.017)
CS	12.441 (0.006)	16.34 (0.012)
FADS2	12.497 (0.006)	14.181 (0.028)
GSS	11.13 (0.011)	18.092 (0.006)
PGD	9.885 (0.020)	16.879 (0.010)

### Distinguishing two subtypes of MAFLD according to ferroptosis-related genes

**Fig. 2:**
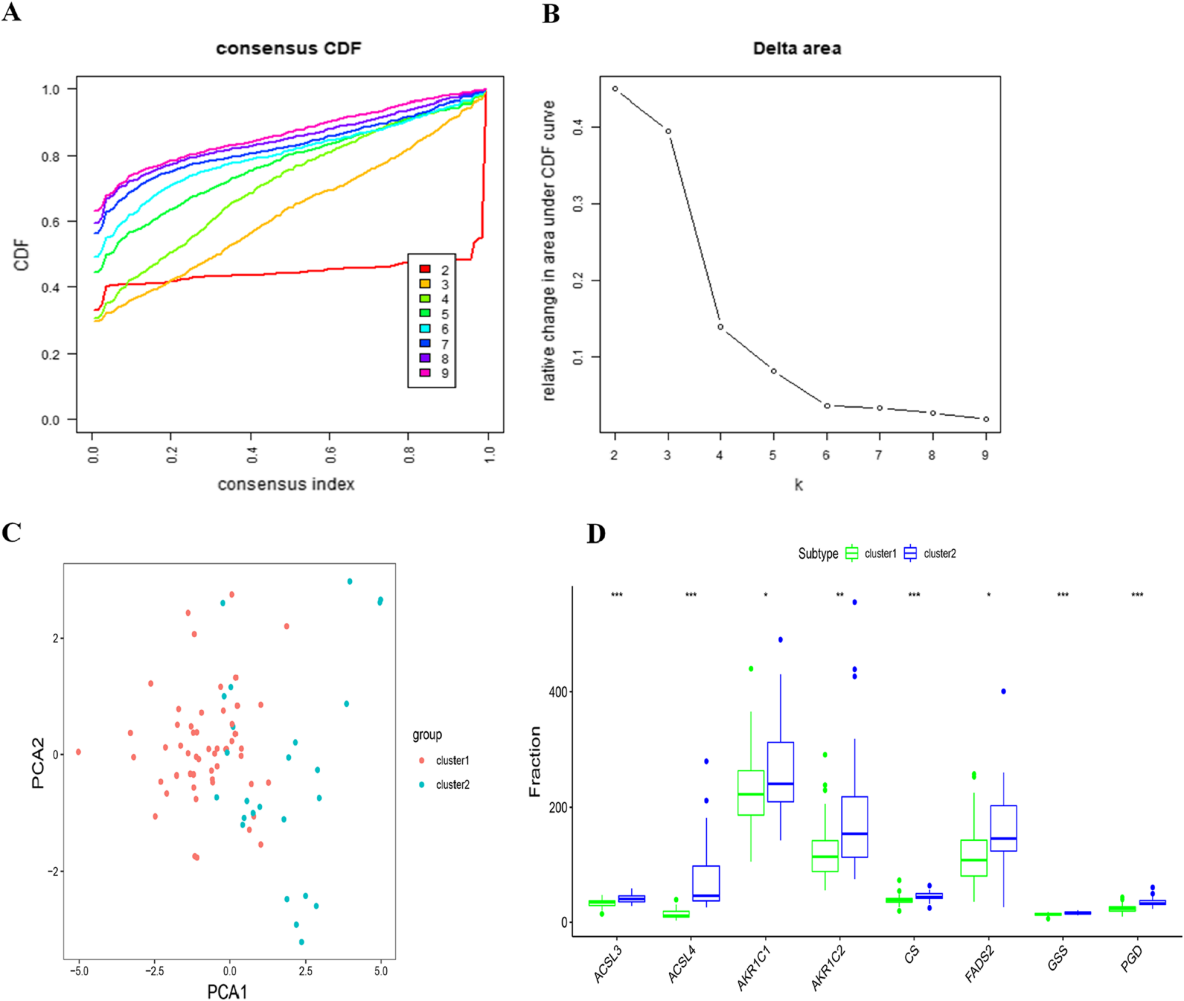
Identification of optimal MAFLD subtypes based on ferroptosis-related genes. **A** The CDF curves are integrals of the probability density function, which completely describes the probability distribution of a real random variable and is established using a consensus clustering approach. CDF curves of consensus scores based on different subtype numbers (k = 2, 3, 4, 5, 6, 7, 8 and 9) are represented in the corresponding colors. **B** The CDF Delta area curve of all samples when k = 2. **C** PCA showed that the two subtypes were clearly distinguished. **D** Eight ferroptosis-related genes were upregulated in the Cluster 2 subtype of MAFLD. CDF, cumulative distribution function; PCA, principal component analysis; MAFLD, metabolic dysfunction-associated fatty liver disease

We performed K-means consensus clustering for 78 patients with MAFLD from k = 2 to k = 9 based on the similarity of expression of ferroptosis related genes. Notably, k = 2 appeared to be an optimal selection. The results were most reliable and stable when patients with MAFLD were divided into two subtypes, with 53 patients in Cluster 1 and 25 patients in Cluster 2 (Additional file 1: Fig. S1). PCA showed that the two subtypes were clearly distinguished. In addition, all 8 ferroptosis-related genes were upregulated in the Cluster 2 subtype of MAFLD (Fig. 2).

### Heterogeneity in the clinical features of the two subtypes of MAFLD

**Fig. 3:**
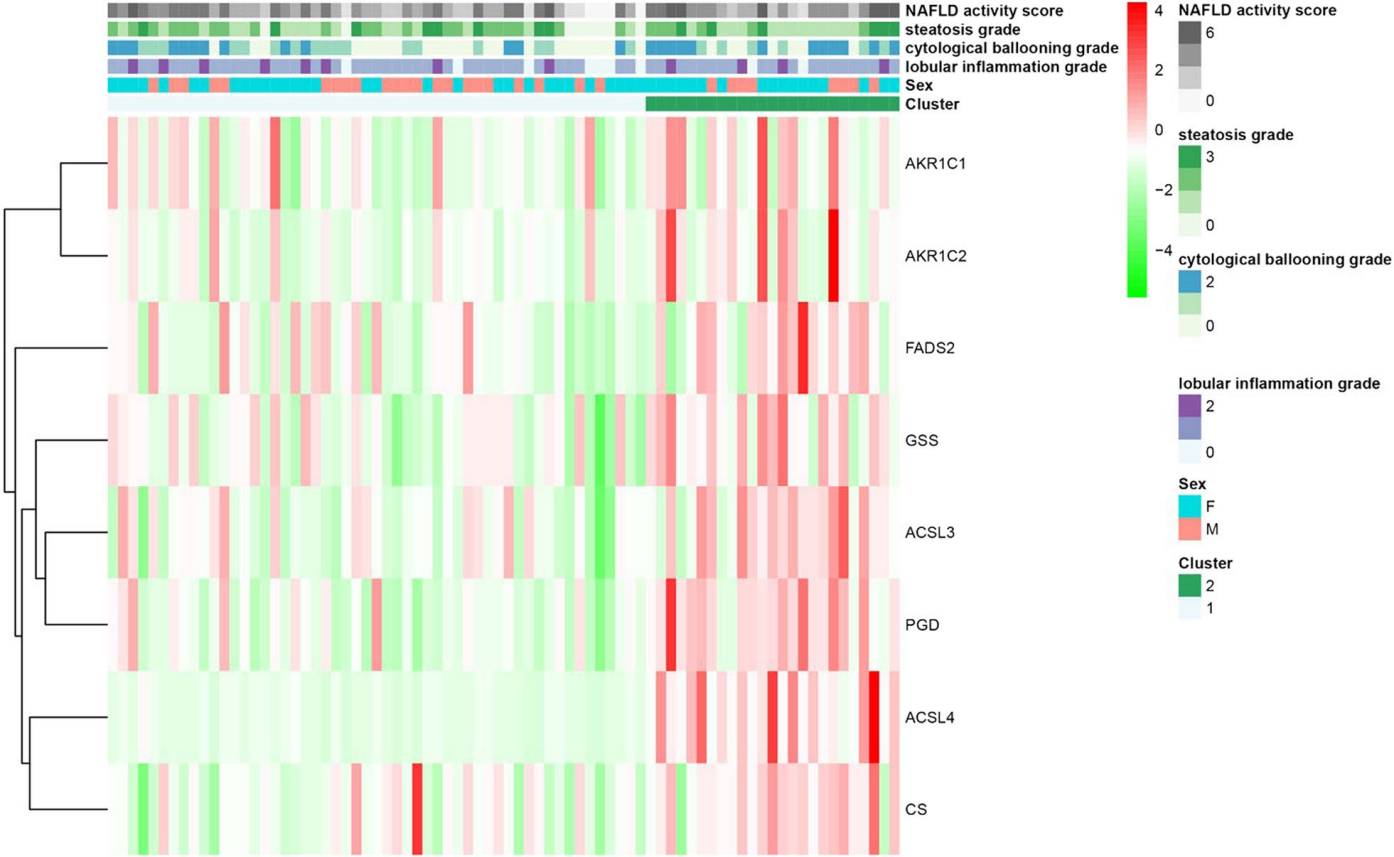
Heterogeneity in the clinical features of the two subtypes of MAFLD. The distribution of 8 ferroptosis-related genes among the 2 subtypes. MAFLD, metabolic dysfunction-associated fatty liver disease

The analysis of the clinical features of the two subtypes revealed significant differences between the two subtypes in hepatic steatosis grade (P < 0.05) and NAS (P < 0.005). Remarkably, 15.1% of patients in Cluster 1 did not have hepatic steatosis. However, all the patients in Cluster 2 had varying degrees of hepatic steatosis, and 24% of them had the highest grade. Meanwhile, regarding NAS, 7.5% of patients had a score of 0, and more than half of the patients scored less than 3 points in Cluster 1. In contrast, the patients in Cluster 2 had higher NAS. Twenty four percent of patients had a score of 6. These data indicated that the clinical features of the patients in Cluster 2 were more serious than those in Cluster 1 (Table 2, Fig. 3).

**Table 2:** Heterogeneity in the clinical features of the two clusters of MAFLD

	Cluster1	Cluster2	*p*
n	53	25	
Sex = M (%)	22 (41.5)	8 (32.0)	0.578
Lobular inflammation grade (%)			0.798
0	7 (13.2)	2 (8.0)	
1	38 (71.7)	19 (76.0)	
2	8 (15.1)	4 (16.0)	
Cytological ballooning grade (%)			0.114
0	23 (43.4)	7 (28.0)	
1	17 (32.1)	6 (24.0)	
2	13 (24.5)	12 (48.0)	
Steatosis grade (%)			0.048
0	8 (15.1)	0 (0.0)	
1	16 (30.2)	13 (52.0)	
2	21 (39.6)	6 (24.0)	
3	8 (15.1)	6 (24.0)	
NAS (%)			0.003
0	4 (7.5)	0 (0.0)	
1	4 (7.5)	1 (4.0)	
2	5 (9.4)	4 (16.0)	
3	15 (28.3)	3 (12.0)	
4	7 (13.2)	9 (36.0)	
5	16 (30.2)	2 (8.0)	
6	2 (3.8)	6 (24.0)

### Heterogeneity in the immune status between the two subtypes

**Fig. 4:**
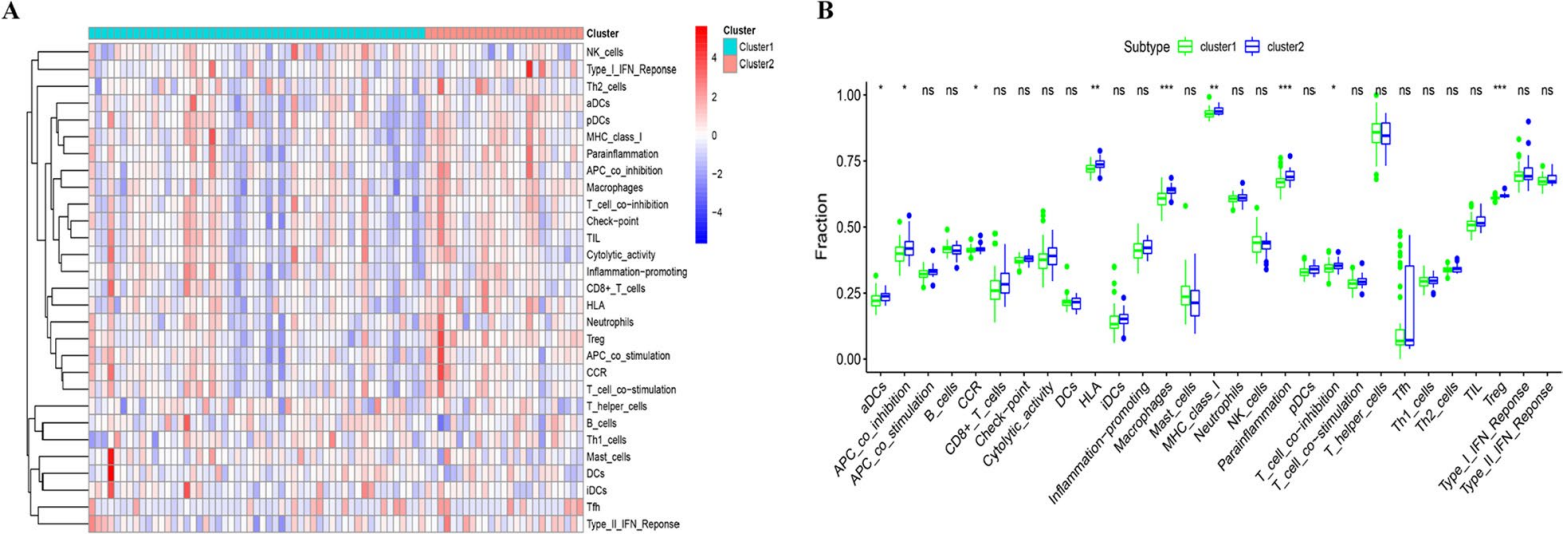
Heterogeneity in the immune status between the two subtypes. **A** The level of immune cell infiltration among subtypes is presented in a heatmap. The enrichment score for every patient was calculated using a single sample Gene Set Enrichment Analysis. **B** Immune cells including aDCs, APC co-inhibition, CCR, HLA, macrophages, MHC class I, parainflammation, T cell co-inhibition and Treg were significantly upregulated in Cluster 2 compared with Cluster 1

We first explored 22 subpopulations of immune cells in each patient using the CIBERSORT algorithm (Additional file 3: Fig. S3). Some previous studies have suggested a link between ferroptosis and immunity [6]. Therefore, we further explored the difference in the enrichment of immune cells between the two subtypes using ssGSEA. After ssGSEA and CIBERSORT analyses, obvious heterogeneity in the immune status between the two subtypes was identified. Compared with Cluster 1, immune cell infiltration was more obvious in Cluster 2 subtypes, where the numbers of aDCs, APC co-inhibition, CCR, HLA, macrophages, MHC class I, parainflammation, T cell co-inhibition and Treg were significantly increased (Fig. 4). Based on these data, the Cluster 2 subtype was characterized by a high level of immune cell infiltration.

### Heterogeneity between the two subtypes by WGCNA

**Fig. 5:**
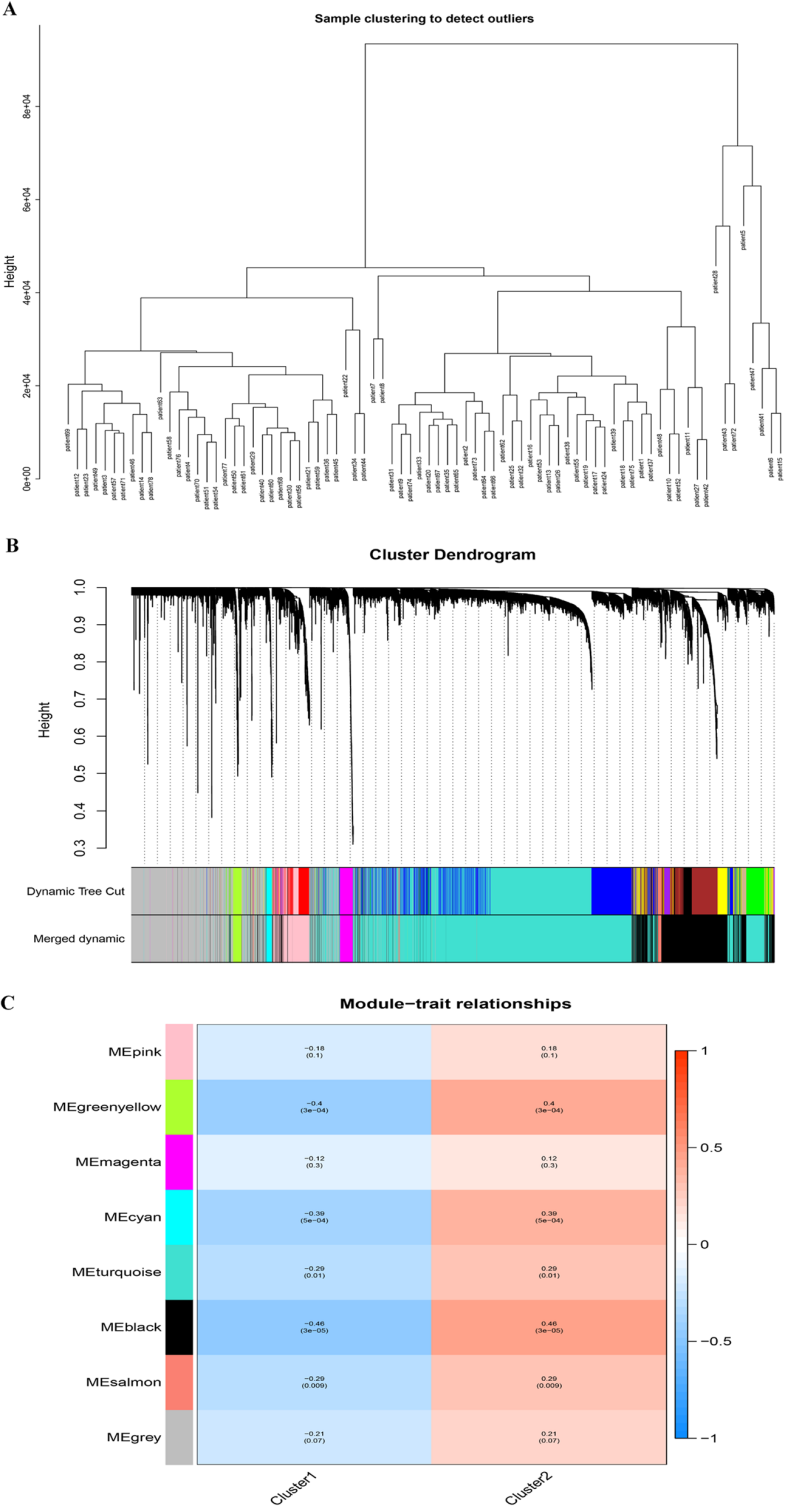
WGCNA between the two subtypes. **A** Sample clustering was conducted to detect outliers. All samples are located in the clusters and met the cutoff thresholds. **B** Modules constructed using WGCNA. **C** The 8 modules were validated and designated with the following colors: black, cyan, green yellow, gray, magenta, pink, salmon and turquoise. Heatmap showing the correlations between the feature vectors of 8 modules and 2 subtypes. WGCNA, weighted gene coexpression network analysis

**Fig. 6:**
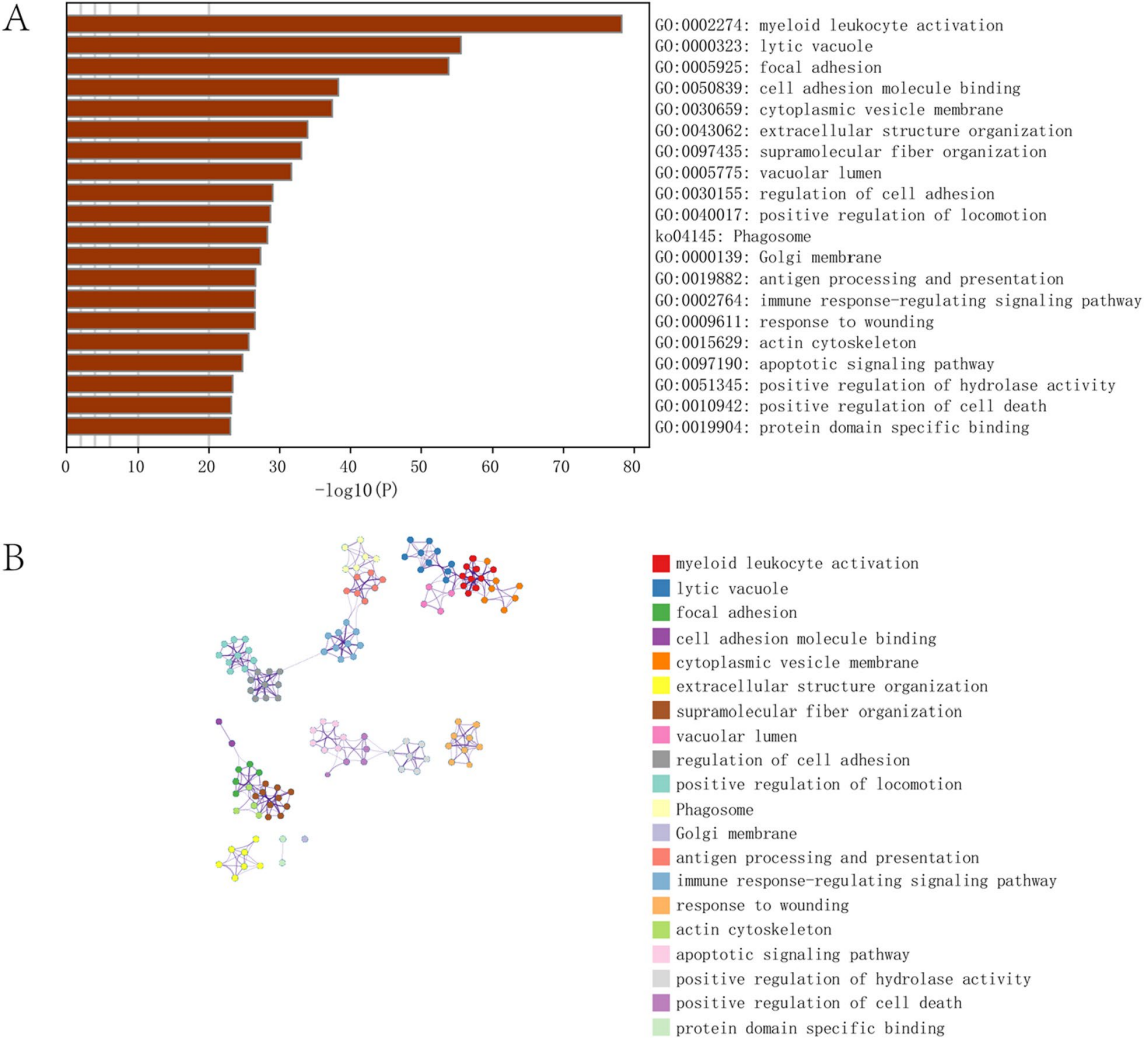
Functional enrichment of module genes related to the phenotype. **A** Gene Ontology enrichment analysis of genes in the black module. **B** The protein–protein interaction network

According to the gene expression data from the patients and the classification of the two subtypes, we performed WGCNA to explore the heterogeneity between the two subtypes. The soft threshold was determined using the function "SFT $powerEstimate". The soft threshold was 9 (Additional file 2: Fig. S2). Then, modules were formed based on a topological overlap matrix. Eight different coexpression modules were obtained, including black (including 1786 genes), cyan (including 90 genes), green yellow (including 150 genes), gray (including 2140 genes), magenta (including 205 genes), pink (including 505 genes), salmon (including 104 genes) and turquoise (including 5020 genes). We further analyzed the relationships between modules and traits and found that the black module was markedly associated with the traits in the two clusters, with a correlation coefficient of ± 0.46 and a P value of 3e−05. Therefore, the black module was selected for subsequent analysis (Fig. 5). Kyoto Encyclopedia of Genes and Genomes and Gene Ontology enrichment analyses were performed for all the genes in the black module to further elaborate the biological functions of genes in this module. The upregulated genes were mainly enriched in myeloid leukocyte activation, antigen processing and presentation and immune response-regulating signaling pathways. Thus, the progression of the two subtypes was associated with immunity, consistent with our expectations. The signal transduction pathways were organized in a network through a series of protein–protein interactions. The protein–protein interaction network is shown in Fig. 6.

### External validation of key ferroptosis related genes

**Fig. 7:**
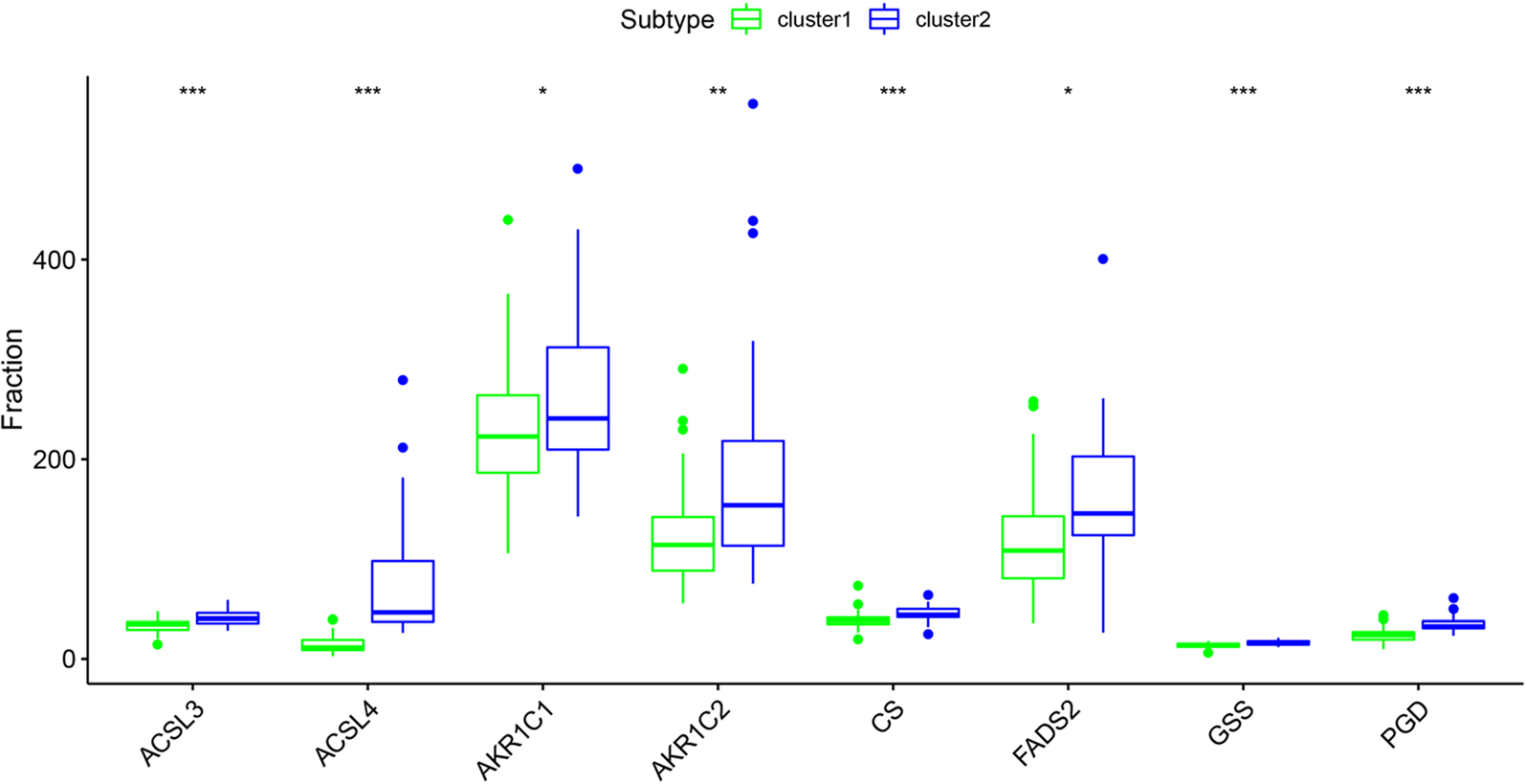
Differences in the expression of key ferroptosis-related genes between the two subtypes in the GSE89632 database. ACSL3, FADS2 and GSS were differently expressed between the two subtypes

**Fig. 8:**
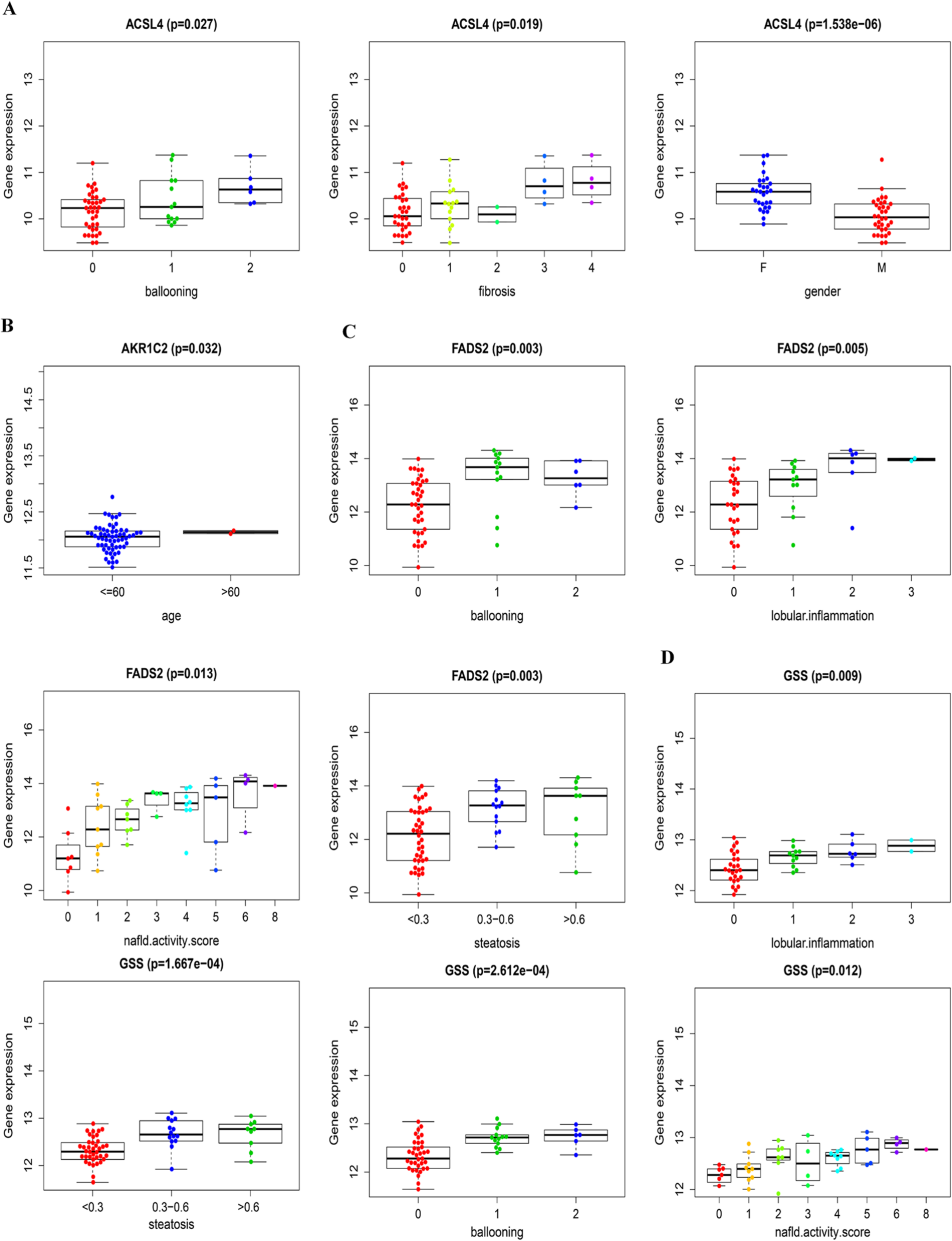
Correlations between key ferroptosis-related genes and clinical features in the GSE89632 dataset. **A** Correlation between ACSL4 expression and clinical features. **B** Correlation between AKR1C2 expression and clinical features. **C** Correlation between FADS2 expression and clinical features. **D** Correlation between GSS expression and clinical features

We downloaded the GSE89632 dataset to verify the stability of the differential expression of ferroptosis-related genes between the two subtypes of MAFLD. We analyzed the clinical information from the validation cohort GSE89632 and tabulated it in Table 3. Consistent with the GSE130970 results, significant differences in hepatic steatosis (P < 0.05) and NAS (P < 0.05) were observed between the two subtypes. ACSL3, FADS2 and GSS were differently expressed between the two subtypes. Compared with subtype 1, the expression of key ferroptosis-related genes, such as FADS and GSS, was significantly increased in subtype 2 (Fig. 7). In addition, ACSL4, AKR1C2, FADS2 and GSS expression were associated with the clinical features (Fig. 8). More research is needed in the future to investigate the potential mechanism underlying their participation in MAFLD.

**Table 3:** Clinical features of the two subtypes of MAFLD in the GSE89632 dataset

	1	2	*p*
n	35	28	
Steatosis (%)			0.047
< 0.3	25 (71.4)	13 (46.4)	
0.3–0.6	7 (20.0)	10 (35.7)	
> 0.6	3 (8.6)	5 (17.9)	
Fibrosis (%)			0.224
0	15 (42.9)	16 (57.1)	
1	10 (28.6)	7 (25.0)	
2	4 (11.4)	2 (7.1)	
3	3 (8.6)	2 (7.1)	
4	3 (8.6)	1 (3.6)	
Lobular inflammation (%)		0.192	
0	14 (40.0)	16 (57.1)	
1	10 (28.6)	6 (21.4)	
2	7 (20.0)	4 (14.3)	
3	4 (11.4)	2 (7.1)	
Ballooning (%)			0.158
0	19 (54.3)	20 (71.4)	
1	11 (31.4)	6 (21.4)	
2	5 (14.3)	2 (7.1)	
NAS (%)			0.025
0	6 (17.1)	3 (10.7)	
1	5 (14.3)	2 (7.1)	
2	5 (14.3)	3 (10.7)	
3	6 (17.1)	2 (7.1)	
4	5 (14.3)	4 (14.3)	
5	3 (8.6)	3 (10.7)	
6	3 (8.6)	4 (14.3)	
7	1 (2.9)	5 (17.9)	
8	1 (2.9)	2 (7.1)	
Age = > 60 (%)	2 (5.7)	0 (0.0)	0.498
Sex = M (%)	19 (54.3)	15 (53.6)	0.955

## Discussion

MAFLD is a common disease worldwide with substantial heterogeneity in the clinical presentation and course. The heterogeneity of patients with MAFLD is regarded as an important obstacle to discovering highly effective drug treatments. Therefore, studies exploring a regulatory analysis that affects heterogeneity and facilitates the elucidation of MAFLD pathogenesis are quite valuable.

In the current study, we revealed that ferroptosis-related genes are closely related to the heterogeneity of MAFLD. Two subtypes of MAFLD were identified by consensus clustering based on the expression of ferroptosis-related genes. The two subtypes exhibited significantly distinct clinical features, immune statuses, biological processes and outcomes. Furthermore, we verified the expression patterns of key ferroptosis-related genes between patients with MAFLD and controls in another dataset. To the best of our knowledge, this study is the first to investigate ferroptosis-related genes in patients with MAFLD using a bioinformatics analysis.

In our current study, we revealed that ferroptosis, clinical features of MAFLD and immune status were associated with each other. These results were consistent with published studies. On the one hand, ferroptosis is involved in the development of MAFLD. For instance, secondary products of lipid peroxidation, such as malondialdehyde and 4-hydroxinonenal are increased in patients with MAFLD due to aberrant oxidative stress. Iron accumulation arising from metabolic aberrations may aggravate the process of MAFLD [13, 14]. On the other hand, ferroptosis induces inflammation. Linkermann A et al. reported that cells undergoing ferroptosis released damage-associated molecular patterns that trigger the innate immune system and induced inflammation [15]. Wenjun Li et al. discovered the initiation of inflammatory responses after cardiac transplantation through ferroptosis and TLR4/Trif-dependent signaling in graft endothelial cells [16]. More importantly, Shinya Tsurusaki et al. showed that hepatic ferroptosis played a critical role in initiating inflammation in individuals with MAFLD [6]. In addition, because we observed differences in immune cell infiltration between the two subtypes and the key module identified by WGCNA was associated with immune pathways, we would like to ascribe the differences in clinical features to different immune microenvironments. Cluster 2, which is defined as the immunoactivated type, has more serious clinical features than Cluster 1, the immunosuppressive type.

An analysis of two datasets revealed that ferroptosis-related genes, such as ACSL3, FADS2 and GSS, played important roles in MAFLD. ACSL3 is an important member of the long chain fatty acyl-CoA synthetase (ACSL) family. ACSLs activate fatty acids through ATP-dependent coenzyme A thioesterification to generate fatty acyl-CoAs that participate in numerous lipid metabolic pathways, including MAFLD [17, 18]. ACSL3 localizes to the endoplasmic reticulum, lipid droplets, *trans*-Golgi network and insulin-containing secretory granules [19–21]. It has been reported that ACSL3 is relevant to ferroptosis. Ubellacker JM et al. reported that oleic acid protected melanoma cells from ferroptosis in an ACSL3-dependent manner [22]. Haarith Ndiaye et al. revealed that the expression of ACSL3 was lower in healthy tissue, but increased in hepatocellular carcinoma and hepatic metastases, which was correlated with ferroptosis [23]. According to Leslie Magtanong et al., exogenous monounsaturated fatty acids strongly inhibit ferroptosis. Importantly, exogenous monounsaturated fatty acids activation required ACSL3 and was independent of lipid droplet formation [24].

FADS2 is a key enzyme involved in the metabolism of n-3 and n-6 polyunsaturated fatty acids, which enable alpha-linolenic acid and linoleic acid to form long-chain polyunsaturated fatty acids and participate in the development of MAFLD. A number of studies show higher FADS2 activity in patients with MAFLD than in normal people [25, 26]. Our result is consistent with these findings. However, the research on FADS2 and MAFLD is limited. Yiqun Jiang et al. knocked down FADS2 in lung cancer cells and found that the level of ferroptosis decreased [27].

GSS is an enzyme important for the synthesis of glutathione, whose depletion results in the initiation and execution of ferroptosis [28]. The antioxidant glutathione exerts a protective effect on the development of MAFLD [29].

This study may have some limitations. First, as a retrospective study including mainly publicly accessible data, we were not able to obtain more demographic and clinical features, such as disease evolution, complications, and individual treatment, to complete more in-depth and longitudinal analyses. Furthermore, considering the limitations of bioinformatics, further studies and experiments should be conducted to verify and investigate the mechanisms of ferroptosis in MAFLD.

## Conclusions

Two highly heterogeneous subtypes of MAFLD with significantly distinct clinical features, biological processes and immune statuses were identified based on the expression of ferroptosis-related genes, which strongly supports the hypothesis that ferroptosis plays an important role in the development of MAFLD. More studies and experiments should be conducted in the future to verify and investigate the mechanisms of ferroptosis in MAFLD.

## Supplementary Information

** Additional file 1: Fig. S1.** Consensus heatmap. Consensus heatmaps for k = 2–9 based on the similarity in the expression of ferroptosis-related genes are shown. A relatively stable partitioning of the samples is observed at k = 2.

**Additional file 2: Fig. S2.** Network topology analysis using various soft thresholding powers for the weighted gene coexpression network analysis. The soft threshold was determined using the function "SFT $powerEstimate". The soft threshold was 9.

**Additional file 3: Fig. S3.** The proportions of immune cells in patients with MAFLD. **A** The proportions of immune cells in each sample are indicated with different colors. The lengths of the bars indicate the levels of the immune cell populations. **B** Correlation matrix for all 22 immune cell proportions. Blue indicates that some immune cells are negatively correlated, and red indicates that some immune cells are positively correlated. The deeper the color, the stronger the correlation (P < 0.05).

## Data Availability

The datasets generated and/or analyzed during the current study are available in the GEO database (https://www.ncbi.nlm.nih.gov/geo/) and PubMed database (https://pubmed.ncbi.nlm.nih.gov/).

## Abbreviations

MAFLD: Metabolic dysfunction-associated fatty liver disease
GEO: GENE EXPRESSION OMNIBUS
ssGSEA: Single sample gene set enrichment analysis
CIBERSORT: Cell-type identification by estimating relative subsets of RNA transcript
WGCNA: Weighted gene coexpression network analysis
NAFLD: Non-alcoholic fatty liver disease
NAS: Non-alcoholic fatty liver disease activity score
PCA: Principal component analysis
ACSL3: Acyl-CoA synthetase long-chain family member 3
ACSL4: Acyl-CoA synthetase long-chain family member 4
AKR1C1: Aldo-keto reductase family 1 member C1
AKR1C2: Aldo-keto reductase family 1 member C2
CS: Citratesynthase
FADS2: Fatty acid desaturase 2
GSS: Glutathione synthetase
PGD: Phosphoglycerate dehydrogenase
