# Supplementary figures and images for: Contribution of classification based on ferroptosis-related genes to the heterogeneity of MAFLD
Source: BMC Gastroenterol. 2022 Feb 10;22:55. doi: 10.1186/s12876-022-02137-9 (PMC8830092; doi:10.1186/s12876-022-02137-9)

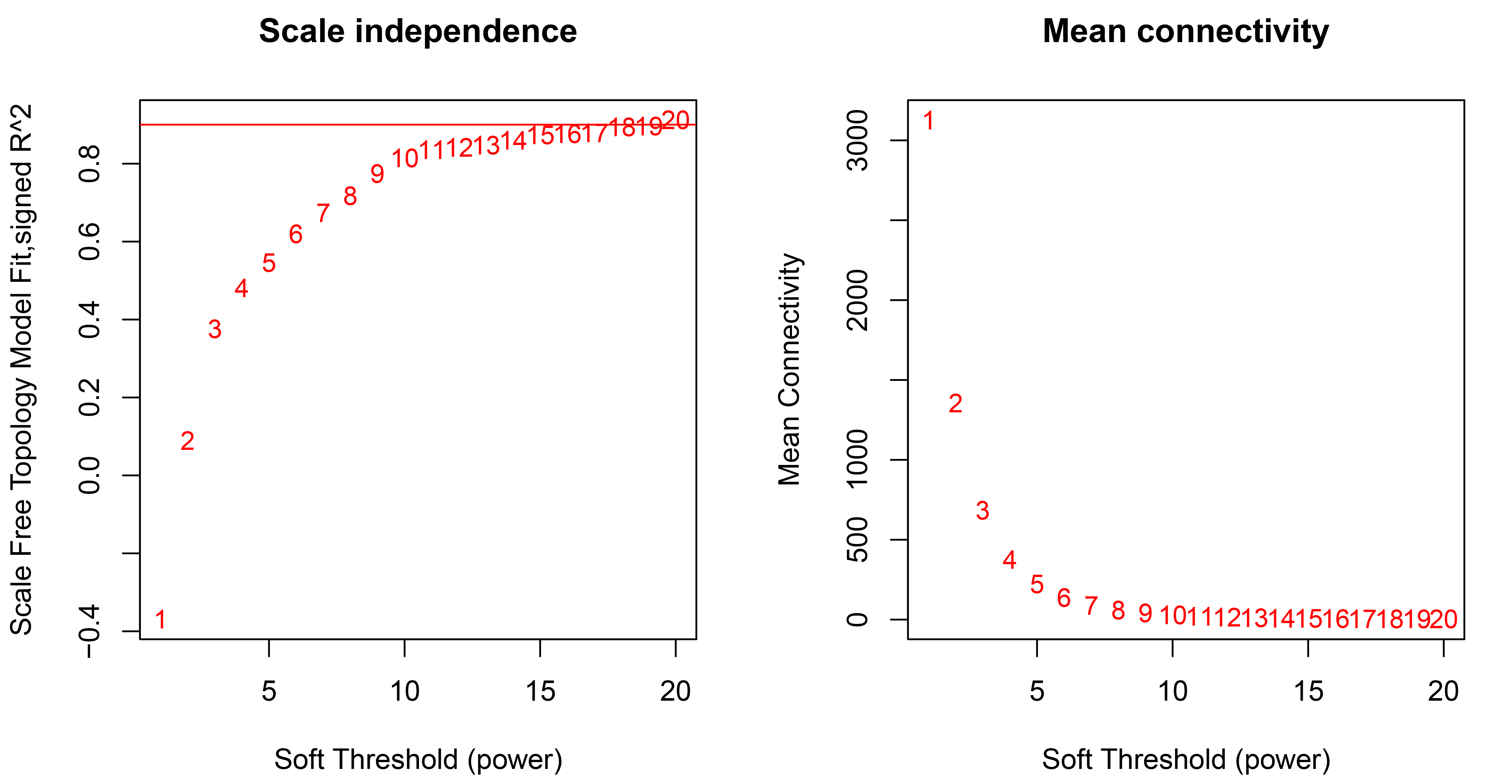

Supplement: Supplementary file 2 — Additional file 2: Fig. S2. Network topology analysis using various soft thresholding powers for the weighted gene coexpression network analysis. The soft threshold was determined using the function "SFT $powerEstimate". The soft threshold was 9. [file 12876_2022_2137_MOESM2_ESM.tif]
